# A Case Report on Implant-Supported Telescopic Prosthesis—A Gerodontic Enhancement

**DOI:** 10.7759/cureus.23558

**Published:** 2022-03-28

**Authors:** Prathibha Saravanakumar, Uma Maheswari, S. Madhan Kumar

**Affiliations:** 1 Prosthodontics, Sri Ramachandra Institute of Higher Education and Research, Chennai, IND

**Keywords:** telescopic prosthesis, implant-supported overdentures, edentulous mandible, rehabilitation, gerodontology

## Abstract

One of the most challenging and demanding endeavors in the field of prosthodontics is the rehabilitation of an edentulous mandible. Implants have been used to support mandibular or maxillary prosthetic rehabilitation. Advantages of using implants are increased retention, increased chewing ability, and also easy access to oral hygiene procedures. For decades, the use of telescopic and conical crowns are in practice to connect natural teeth and not many cases have been reported in the literature of telescopic crowns placed on implants to support the prosthesis. Telescopic crowns provide the best possible distribution of force to the abutment teeth and can improve the patient’s oral health-related quality of life. The use of telescopic crowns as attachments for implant-supported overlying prostheses may be a viable treatment option for edentulous mandible. The functional and the aesthetic enhancement by telescopic retainers are favorable features for many challenging situations.

This case report discusses a 60-year-old male, who reported to the department of prosthodontics with three implants and two bi-cortical screws that were placed in a completely edentulous mandibular arch by a private practitioner one year back and this situation was prosthodontically managed by us with an implant-supported telescopic removable prosthesis.

## Introduction

The fundamental objective in the field of prosthodontics is to return the patient to normal function as possible. There is a wide range of prosthetic treatment options in the rehabilitation of an edentulous patient. Simple conventional removable dentures, fixed complete dentures, overdentures, and/or dental implants can be used for the restoration of lost tooth structure.

The basic overdenture concept is to preserve the residual soft and hard tissues. Like Keshk et al. [1] described in their article, the frictional fit between the primary and the secondary copings in telescopic attachments is excellent and offers enhanced retention. Augmentation of the alveolar ridge, increasing the vestibular depth to provide anchorage for an all implant-supported prosthesis or mucosa, and implant-supported overdentures are viable treatment alternatives. The introduction of implants has improved the quality of life for edentulous patients. Implant-supported overdentures have been shown to provide a successful long-term outcome, particularly when used to restore the edentulous mandible. A conventional complete mandibular denture is less favorable than a complete maxillary denture in terms of retention. Kourtis et al. [2] have also mentioned in their research article that the implant-supported overdentures and telescopic copings have multiple clinical advantages in the rehabilitation of edentulous mandible for both the clinician and the patient. ELsyad et al. [3] have mentioned in their article that telescopic attachments allow freedom in implant placement with a self-finding mechanism that facilitates easy prosthesis insertion.

Here, presenting a case report on prosthetic rehabilitation of a completely edentulous patient using a mandibular implant-supported removable prosthesis and a conventional maxillary removable complete denture. Hakkoum et al. [4] mentioned in their research article that dentist preference and clinical situations decide the selection of the design whether it is to be a rigid or resilient type.

## Case presentation

**Figure 1:**
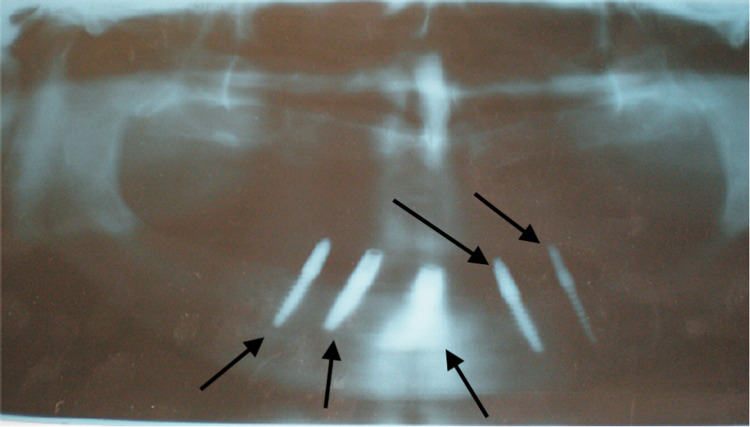
Orthopantomograph with Bi-cortical Screws and Implants

A 60-year-old male patient reported a complaint of completely missing teeth in the upper and lower arch, which led to difficulty in masticating food and problems with phonetics. The patient had no medical complications (diabetes, hypertension) and was mainly worried about his compromised facial appearance. On intra-oral examination, all teeth were found to be missing in the maxillary and mandibular arch. The residual ridge was moderately resorbed. The mandibular arch also showed the presence of five implants in A, B, C, D, and E. However, An orthopantomograph (OPG) (Siemens dental system, Bensheim, Germany) revealed that two were bi-cortical screws and the remaining three were implanted (only three of the five screwed structures were actually dental implants, capable of load-bearing, whereas the remaining two were bi-cortical screws) (Figure 1).

**Figure 2:**
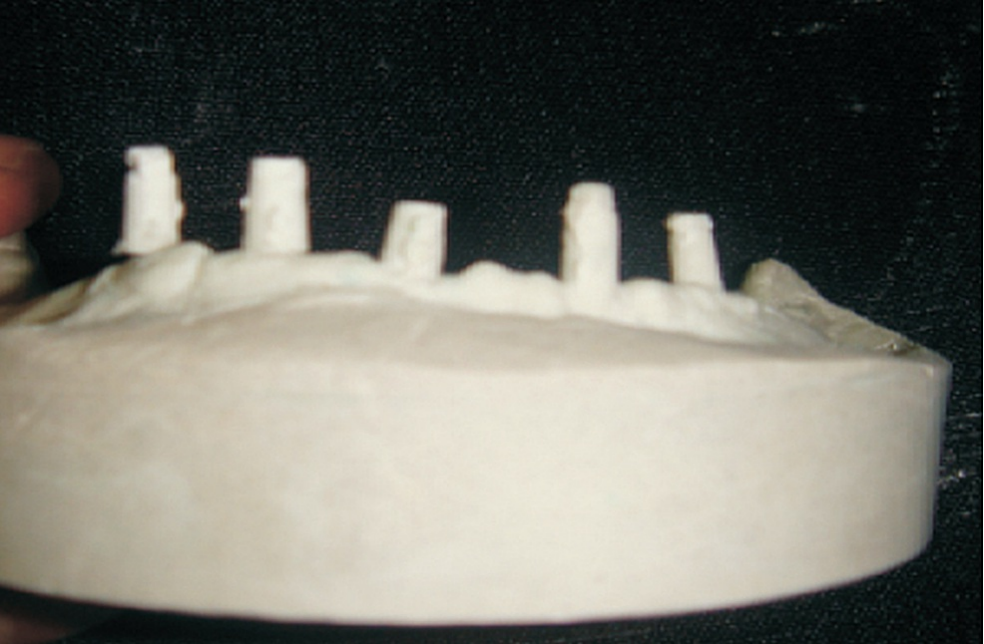
Orthokal Study Models

The maxillary ridge had sufficient bone quantity and quality. Based on the patient’s economic status, a conventional complete denture prosthesis was planned for the maxillary arch. The mandibular ridge however posed a unique problem. Only three out of five abutments in the mandibular arch were capable of load-bearing. The remaining two were bi-cortical screws that could not be used for bearing masticatory loads. Any treatment plan for this particular situation would have to take into account the presence of these five abutments and at the same time understand the load-bearing capacity of each. After careful deliberation, it was decided to fabricate a mandibular implant-supported telescopic prosthesis for this particular patient. The patient’s history was thoroughly recorded. Maxillary and mandibular study models were made with Orthokal (Kalabai Karson Pvt. Ltd, Mumbai) (Figure 2).

The radiographic evaluation showed stable bone levels around all implants. Once the evidence of osseointegration was established the treatment plan was carried out as planned. Maxillary preliminary impression was made with impression compound (DPI Pinnacle, Bombay Burmah Trading Corporation, India) and the mandibular impression was made in reversible hydrocolloid impression material (Algitex, Bombay Burmah Trading Corporation, India). Casts were fabricated by pouring these impressions with dental stone (Kalabhai Dental PVT. LTD, Kalstone). All the five abutments were prepared intraorally to create a common path of insertion and a second impression was made using additional silicone impression material (Dentsply Aquasil and Coltene Affinis set). Casts were poured and five separate metal copings were fabricated over three implants and two bi-cortical screws and the milled copings were cemented with polycarboxylate luting cement (Carbaco, Voco, Germany).

**Figure 3:**
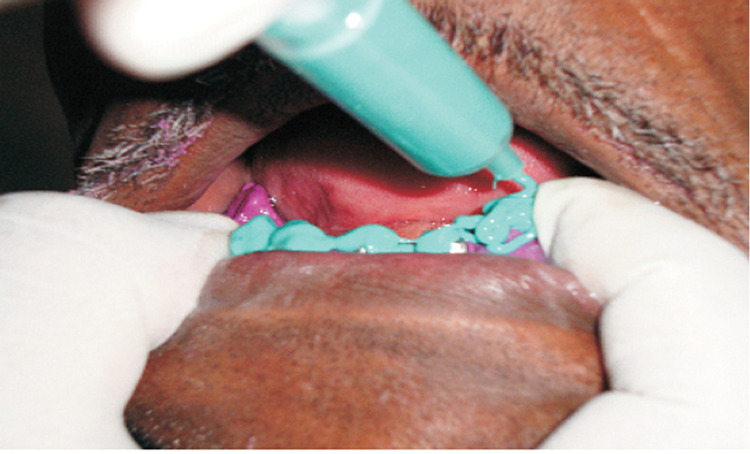
Mandibular Impressions with Putty and Light body

**Figure 4:**
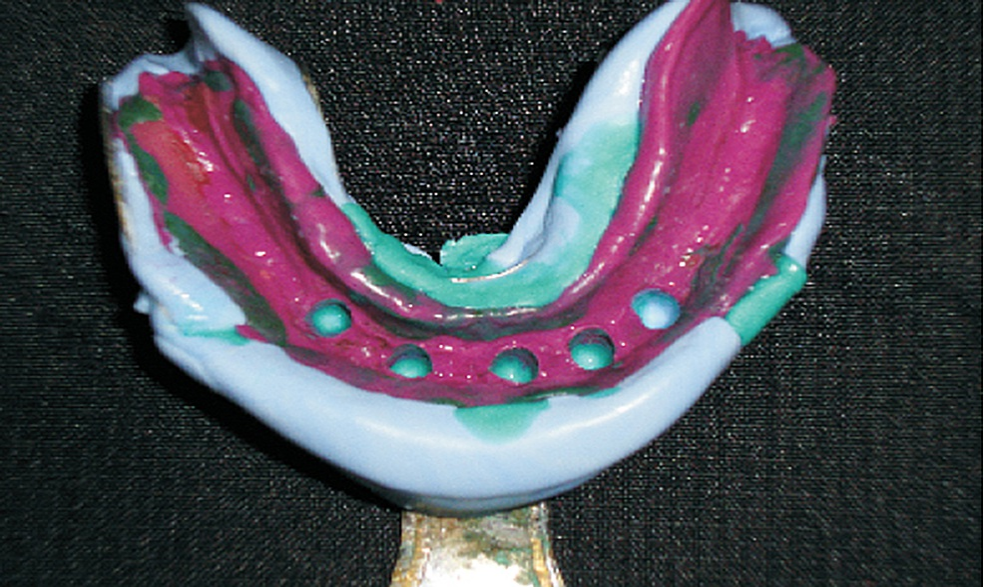
Final Impressions For Construction Of Telescopic Prosthesis

A maxillary and mandibular auto polymerizing acrylic resin (DPI RR COLD CURE, DPI, India) special tray was fabricated and a conventional border molding was done with a low fusing green stick compound (DPI Pinnacle, India) (Figure 3). For maxilla, the final impression was made with medium body impression material and for mandible, a putty border molding was done with a wash impression in the light body (Aquasil Light Body, Dentsply) (Figure 4).

**Figure 5:**
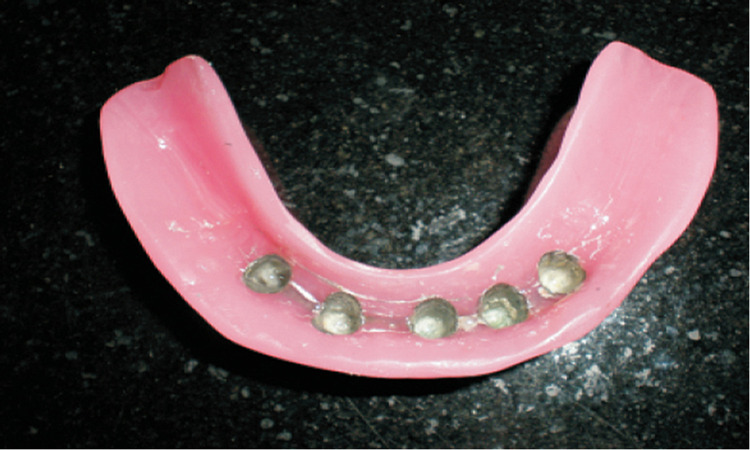
Mandibular Telescopic Prosthesis—Excellent Retention

Then a cast was poured in which a second metal framework was fabricated. This metal framework resembled the framework of a five-unit anterior bridge. It consisted of five metal copings similar to the first set joined together by connectors (Figure 5). The dimensions of the connectors were approximately 3 mm in height and 2 mm in width. The master cast was made in which the wax occlusal rims (Modelling Wax, India) were fabricated. The maxillary and mandibular spatial relationship were recorded and trial insertion is done. The trial prosthesis outcome was quite satisfactory. Finally, the prosthesis was fabricated with heat cure acrylic polymerizing resin (Trevalon, Dentsply).

**Figure 6:**
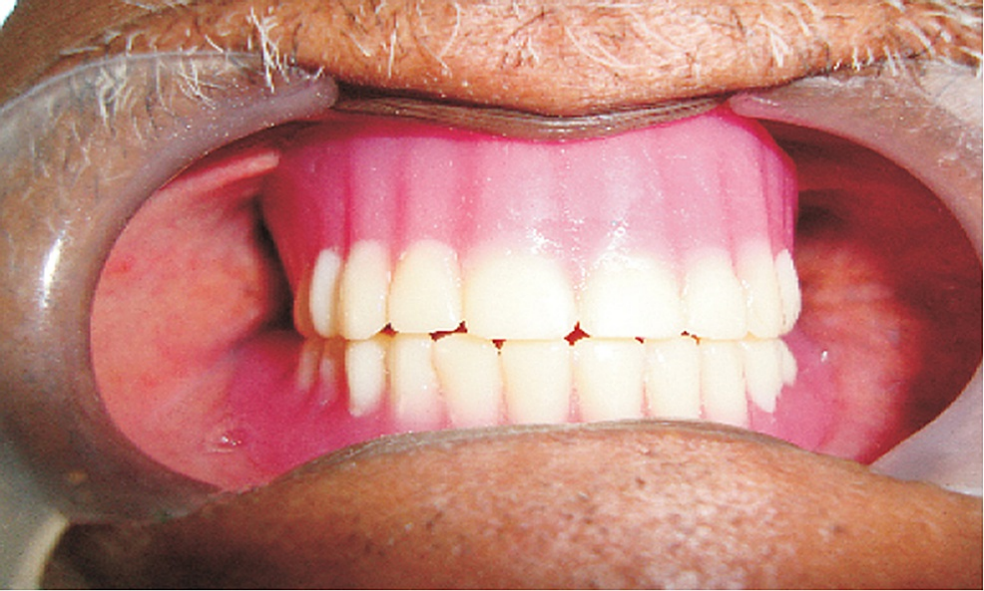
Completed Telescopic Prosthesis

The maxillary and the mandibular removable prosthesis were inserted and minor occlusal adjustments were done (Figure 6). The mandibular prosthesis had excellent retention due to the telescopic fitting of the secondary metal framework (in the undersurface of the prosthesis) with the primary copings (cemented intraorally). The patient was put on regular recall and maintenance. The clinical assessment showed good results.

## Discussion

Removal of teeth leads to phenomenal residual ridge resorption and also causes bone loss which is rapid, progressive, irreversible, and inevitable. On observation, bone maintenance is seen around standing teeth and implants. Residual ridge resorption is more marked in the lower arch and has a rapid spread. As ELsyad et al. [5] described in their article, Osseo-integrated dental implants stabilizing overdentures solve problems with mandibular alveolar ridge atrophy and also avoid complications of vestibuloplasty and ridge augmentation. In edentulous patients, the implant-retained tissue-supported prosthesis is an advanced method for rehabilitation of mandible with poor mucosal and tissue support. Choice of single or several treatment options with implants has been described for mandibular edentulous patients. Like Rinke et al. [6] mentioned in his article, to reduce the risk of abutment overloading double crowns with clearance fit, resilient telescopic crowns have been developed. Similarly, the availability of bone satisfies the functional and aesthetic demands of the patient.

Abutment teeth in telescopic overdentures are subjected to endodontic and periodontal therapy and covered with primary cast copings. Secondary cast copings provide support and frictional retention and are placed over the primary copings and incorporated as an integral part of the prosthesis. The telescoping between metallic copings directly with acrylic channels in the base has its own merits. Arnold et al. [7] had concluded in their study that the highest retention forces were evident with telescopic crowns which had more extended surface contact such as double crowns. The telescopic units had primary gold copings and a set of secondary cast copings attached to the overdenture for stress reduction. Mahana et al. [8] had managed to conclude in their study that in severely atrophied ridges, an attractive treatment option that avoids complications of ridge augmentation and vestibuloplasty would be two implants stabilizing a mandibular overdenture. Telescopic crowns were used as retainers for removable partial dentures in the early phase. Dabrowa et al. [9] mentioned in their study that telescopic crowns which are found in patients with residual and reduced dentition provide anchorage on single abutments and direct the occlusal forces on the long axis of the abutment teeth which in turn aids in longer retention in the alveolar bone. A recent addition to the array of unsplinted attachment systems is the telescopic attachments. The attachment assembly is made up of a primary coping (Patrix) cemented to the implant and a second rigid metal framework (Matrix) that is contained within the prosthesis framework.

The attachment can be of rigid design or resilient design depending on the degree of fit between the two copings. Similar to the majority of ball attachments, the retention of a telescopic attachment system is also obtained through the frictional contact between its components. Clinicians often base their selection of attachment system for mandibular implant-retained prosthesis empirically on their presumed retentive qualities; this is evident in the mandibular implant-retained telescopic prosthesis, where adequate retention has been correlated with improved levels of patient satisfaction. In geriatric patients with marked mandibular atrophy, anchorage and prosthetic stabilization to horizontal forces with resilient telescopic crowns constitute a feasible alternative for retention. Recent clinical publications of implant outcomes using telescopic crowns have confirmed their successful long-term use as an alternate retention modality.

The technical complication with telescopic attachments was significantly lesser when compared to other attachments. When horizontal forces become predominant, the secondary crowns release from the sleeve copings and so the cross-arch torque, which often causes cementation failures, is reduced. Peri-implant conditions and implant success were similar between ball attachments and telescopic crowns. Arunraj et al. [10] mentioned in their study that on the whole, telescopic overdentures have the advantage of providing effective retention, stability, support, and proprioception than a conventional removable denture. The advantage of implant-supported telescopic overdentures also works with increased chewing efficiency and phonetics.

## Conclusions

Edentulous patients display moderate to severe dysfunction, as measured by both subjective and objective physiologic criteria. Osseo-integrated dental implants provide a total solution for the individual’s denture-related problems. A philosophical shift in dentistry can lead to mandibular implant-supported telescopic prosthesis becoming the standard treatment of edentulous patients. Telescopic prosthesis reduces the treatment time and expense and is suited to the lower socioeconomic status of many edentulous patients. The advantages of both fixed and removable restorations can be achieved with a telescopic prosthesis. Their design and configuration vary depending on whether retention, stability, and support are given for the patient.

Clinical skills and experience are required for the fabrication of telescopic prostheses. Chair time taken for the procedure was 3 weeks though, time-consuming clinical procedures combined with complex and expensive laboratory procedures contribute to the high cost of treatment.

This case report gives a detailed explanation of the rigidity and the resiliency of the primary and the secondary components of the telescopic prosthesis and is applied according to the prosthodontic requirements and clinical assessment.

## References

[1] Keshk AM, Alqutaibi AY, Algabri RS, Swedan MS, Kaddah A (2017). Prosthodontic maintenance and peri-implant tissue conditions for telescopic attachment-retained mandibular implant overdenture: systematic review and meta-analysis of randomized clinical trials. Eur J Dent.

[2] Kourtis S, Madianos P, Patras M, Andrikopoulou E (2018). Rehabilitation of the edentulous mandible with implant-supported overdentures on telescopic abutments and immediate loading. A controlled prospective clinical study. J Esthet Restor Dent.

[3] ELsyad MA, Soliman TA, Khalifa AK (2018). Retention and stability of rigid telescopic and milled bar attachments for implant-supported maxillary overdentures: an in vitro study. Int J Oral Maxillofac Implants.

[4] Hakkouma MA, Wazir G (2018). Telescopic denture. Open Dent J.

[5] ELsyad MA, Denewar BA, Elsaih EA (2018). Clinical and radiographic evaluation of bar, telescopic, and locator attachments for implant-stabilized overdentures in patients with mandibular atrophied ridges: a randomized controlled clinical trial. Int J Oral Maxillofac Implants.

[6] Rinke S, Schneider L, Schulz X, Wiedemann V, Bürgers R, Rödiger M (2019). Overdentures borne on less than four abutments with telescopic crowns: 5-year results of a retrospective clinical study. Clin Oral Investig.

[7] Arnold C, Schweyen R, Boeckler A, Hey J (2020). Retention force of removable partial dentures with CAD-CAM-fabricated telescopic crowns. Materials.

[8] Mahanna FF, Elsyad MA, Mourad SI, Abozaed HW (2020). Satisfaction and oral health-related quality of life of different attachments used for implant-retained overdentures in subjects with resorbed mandibles: a crossover trial. Int J Oral Maxillofac Implants.

[9] Dąbrowaa T, Wcisło A, Majstrzyk W, Niedziałkowski P, Ossowski T, Więckiewicz W, Gotszalk T (2021). Adhesion as a component of retention force of overdenture prostheses-study on selected Au based dental materials used for telescopic crowns using atomic force microscopy and contact angle techniques. J Mech Behav Biomed Mater.

[10] Arunraj D, Gnanam P, Chander GN (2021 ). Prosthodontic rehabilitation of a patient with missing teeth and loss of vertical dimension using telescopic overdentures. Contemp Clin Dent.

